# Bioartificial Heart: A Human-Sized Porcine Model – The Way Ahead

**DOI:** 10.1371/journal.pone.0111591

**Published:** 2014-11-03

**Authors:** Alexander Weymann, Nikhil Prakash Patil, Anton Sabashnikov, Philipp Jungebluth, Sevil Korkmaz, Shiliang Li, Gabor Veres, Pal Soos, Roland Ishtok, Nicole Chaimow, Ines Pätzold, Natalie Czerny, Carsten Schies, Bastian Schmack, Aron-Frederik Popov, André Rüdiger Simon, Matthias Karck, Gabor Szabo

**Affiliations:** 1 Department of Cardiac Surgery, Heart and Marfan Center - University of Heidelberg, Heidelberg, Germany; 2 Department of Cardiothoracic Transplantation & Mechanical Circulatory Support, Royal Brompton and Harefield NHS Foundation Trust, Harefield Hospital, London, United Kingdom; 3 Department of Clinical Science, Intervention and Technology, Advanced Center for Translational Regenerative Medicine, Karolinska Institutet, Stockholm, Sweden; 4 Heart and Vascular Center, Semmelweis University, Budapest, Hungary; 5 2^nd^ Department of Pathology, Semmelweis University, Budapest, Hungary; Harefield Hospital, United Kingdom

## Abstract

**Background:**

A bioartificial heart is a theoretical alternative to transplantation or mechanical left ventricular support. Native hearts decellularized with preserved architecture and vasculature may provide an acellular tissue platform for organ regeneration. We sought to develop a tissue-engineered whole-heart neoscaffold in human-sized porcine hearts.

**Methods:**

We decellularized porcine hearts (n = 10) by coronary perfusion with ionic detergents in a modified Langendorff circuit. We confirmed decellularization by histology, transmission electron microscopy and fluorescence microscopy, quantified residual DNA by spectrophotometry, and evaluated biomechanical stability with ex-vivo left-ventricular pressure/volume studies, all compared to controls. We then mounted the decellularized porcine hearts in a bioreactor and reseeded them with murine neonatal cardiac cells and human umbilical cord derived endothelial cells (HUVEC) under simulated physiological conditions.

**Results:**

Decellularized hearts lacked intracellular components but retained specific collagen fibers, proteoglycan, elastin and mechanical integrity; quantitative DNA analysis demonstrated a significant reduction of DNA compared to controls (82.6±3.2 ng DNA/mg tissue vs. 473.2±13.4 ng DNA/mg tissue, p<0.05). Recellularized porcine whole-heart neoscaffolds demonstrated re-endothelialization of coronary vasculature and measurable intrinsic myocardial electrical activity at 10 days, with perfused organ culture maintained for up to 3 weeks.

**Conclusions:**

Human-sized decellularized porcine hearts provide a promising tissue-engineering platform that may lead to future clinical strategies in the treatment of heart failure.

## Introduction

Heart transplantation is the definitive treatment for end-stage heart failure, but is limited by donor organ shortage and waiting-list mortality. Whereas mechanical circulatory support mandates anticoagulation with its inherent risks, heart transplant recipients must live with the “necessary evil” of lifelong immunosuppression and invasive surveillance studies, often begetting hypertension, diabetes, renal failure, malignancy and other sequelae of chronic immunosuppression [1], [2]. A potential solution is a bioartificial or “tissue-engineered” heart. If whole hearts can be decellularized while preserving 3D geometry and vasculature, the resulting scaffold may provide an architectural skeleton for whole-organ tissue engineering. However, due to the density, mass, and 3D architecture of most whole organs such as the heart, liver, and kidney, traditional decellularization methods like immersion-agitation are ineffective at removing cellular material [3]. Similarly, tissue-engineering methods used for heart valves [4]–[6] cannot be simply extended to myocardium because of its biological complexity- native myocardium is a dense, highly vascular tissue with nearly one capillary per cell and thickness of up to one centimeter; diffusion cannot support tissues thicker than 100 microns and would be insufficient to support a thick cardiac tissue–engineered construct [7]. Thus, tissue-engineered myocardium requires elaborate ex vivo culture conditions.

Whereas considerable progress has been made with rat hearts [7]–[9], experiments with human-sized porcine hearts lag much behind [10], [11]. We present the first experimental prototype of a tissue-engineered porcine whole-heart, with perfused organ culture in a bioreactor and formation of myocardium that generates intrinsic electrical activity.

## Material and Methods

### Procurement of whole porcine hearts

32 porcine hearts (approximate weight 300 g) were procured with aseptic precautions from adult female Large-White-Landrace crossbred pigs (30–45 kg). All animals received humane care in compliance with the Principles of Laboratory Animal Care formulated by the National Society for Medical Research and the Guide for the Care and Use of Laboratory Animals prepared by the Institute of Laboratory Animal Resources and published by the National Institutes of Health (NIH Publication No. 86–23, revised 1996). All procedures followed the European Agreement of Vertebrate Animal Protection for Experimental Use (86/609). This investigation was reviewed and approved by the ethical committee for animal experimentation at the University of Heidelberg (35-9185.82/A-27/07).

### Anesthetic and Surgical Procedures

Intravascular access was secured via a superficial ear vein of the animal, as described previously [12]. After intramuscular injection of 4 mg Azaperone (Stresnil, Janssen, High Wycombe, UK) and 0.01 mg/kg Fentanyl, 3–4 mg/kg Hypnomidate was administered intravenously, followed by intubation and ventilation with 40% FiO_2_. Muscle relaxation was achieved with Pancuronium (0.3 mg/kg/h). After systemic heparinization and median sternotomy, we arrested the heart with antegrade cardioplegia using cold (4°C) Custodiol solution (HTK solution, Dr. Franz Köhler Chemie GmbH, Alsbach-Hähnlein, Germany). We transected the venae cavae, pulmonary veins, pulmonary artery and thoracic aorta, and explanted the heart.

### Perfusion Decellularization Circuit

The modified Langendorff decellularization model comprised a perfusion circuit and a pressure control module, as described previously [11]. All components were sterilized by conventional autoclaving and connected with 3/8” silicon tubes (Maquet, Rastatt, Germany). We cannulated the aorta with a 25–27 mm Teflon cannula for antegrade coronary perfusion, powered by a roller pump (Stöckert, München, Deutschland) controlled via a pressure transducer (Medex Smith Medical, Kent, United Kingdom). A computer system (Engineo, Mainz, Germany), continuously recorded the perfusion pressure. We used a heat exchanger (D720 Helios C, Dideco, Mirandola, Italy) for perfusate temperature-control, and interposed an airtrap (Gambro Medical Line, Hechingen, Germany) to ensure air-free perfusate. The returning (outflow) perfusate was collected into a reservoir (Maquet AR 28150, Rastatt, Germany), with total circuit volume of 2500 mL.

We perfused the hearts at a constant perfusion pressure of 100 mmHg with a warm (37°C) solution of 4% sodium dodecyl sulfate (SDS) in phosphate-buffered solution (PBS, Sigma, Munich, Germany) at 2 L/min for 12 h, intermittently washing the hearts with PBS for 15 min every 3 h to remove residual substances. We then perfused antibiotic-containing PBS enriched with 100 µg/mL penicillin-streptomycin (Biochrom, Berlin, Germany) through the hearts at 1.5 L/min for 24 h to remove any residual detergent and cell debris, followed by overnight incubation in Dulbecco's Modified Eagle Medium (DMEM) (Invitrogen, Darmstadt, Germany) at 4°C.

### Evaluation of Biomechanical Stability

For the measurement of mechanical stability n = 6 porcine hearts in each experimental group (native vs. decellularized) were used. We introduced a latex balloon catheter into the left ventricle (LV) via the aorta and connected it to a precision calibrated syringe for administration and withdrawal of fluid. We then advanced a Millar micromanometer (Millar Instruments, Inc, Houston, TX, USA) into the LV via the apex, and measured the LV pressure at different LV volumes.

### DNA Quantification

We processed sections from seven different regions of the heart (apex, LV, right ventricle, septum, papillary muscle, right and left atrium) for spectrophotometric quantification to determine the concentrations of residual DNA in the decellularized group and compared these to controls. Samples (approximately 10–15 mg) were normalized according to equivalent dry weight in mg. The DNA content was subjected to standard silica-membrane-based purification (QIAamp DNA Mini Kit, Qiagen, Basel, Switzerland) before quantification by spectrophotometry.

### Glycosaminoglycan and Elastin Analysis

Total sulfated glycosaminoglycans and cross-linked elastin within the decellularized hearts were determined by a commercial kit (Biocolor, Carrickfergus, United Kingdom) as per the manufacturer's instructions. Lyophilized tissue samples of left and right ventricles from both native and decellularized hearts were digested with papain for the glycosaminoglycan and elastin assay. All values were adjusted to 1 mg dry tissue weight for comparison.

### Transmission Electron Microscopy (TEM)

Tissue specimens of the aortic valve of the decellularized porcine hearts were fixed in 2.5% glutaraldehyde and embedded in Epon resin (PELCO Eponate 12 Kit, 18010, Ted Pella, Inc, Redding, CA, USA). Standard ultrathin sections were prepared, and TEM performed using a Zeiss analytical EM 902 (Zeiss, Oberkochen, Germany) microscope coupled with a Pro-Scan digital camera (Tröndle, Munich, Germany).

### Histology and Immunofluorescence

Hearts were fixed in 10% formalin, embedded in paraffin and sectioned into 5 µm sections. Heart tissue was stained with Masson's trichrome stain (Fomori, procedure HT10, Sigma-Aldrich) to distinguish the cells from the surrounding connective tissue. 4′,6-diamidino-2-phenylindole (DAPI, Sigma-Aldrich Inc, Munich, Germany) and hematoxylin/eosin (H&E) stains were used for initial inspection for remnant nuclear structures. Modified Movat's Pentachrome (Mastertechs, Lodi, CA, USA) stain was used to visualize extracellular matrix components (ECM) such as collagen, elastin and glycosaminoglycans. Herovici stain (American MasterTech, Lodi, USA) was used to identify collagen types I and III in the newly deposited matrix. PECAM-1 (Polyclonal Rabbit IgG, Santa Cruz Biotechnology, Heidelberg, Germany) fluorescent immunohistochemistry [11], [13] was employed for identification of endothelial cells following reseeding with HUVEC. Stained samples from both decellularized and control (native-heart) groups were examined histologically, and the degree of re-endothelialization and preservation of ECM evaluated (blind analysis) by two independent pathologists. The sections were analyzed by routine bright field and fluorescence microscopy (Olympus Optical Co, BX 51 and CKX 41 microscope). Images were acquired with the CellA Soft Imaging System (Olympus Soft Imaging Solutions, Münster, Germany).

### Whole-heart bioreactor

**Figure 1:**
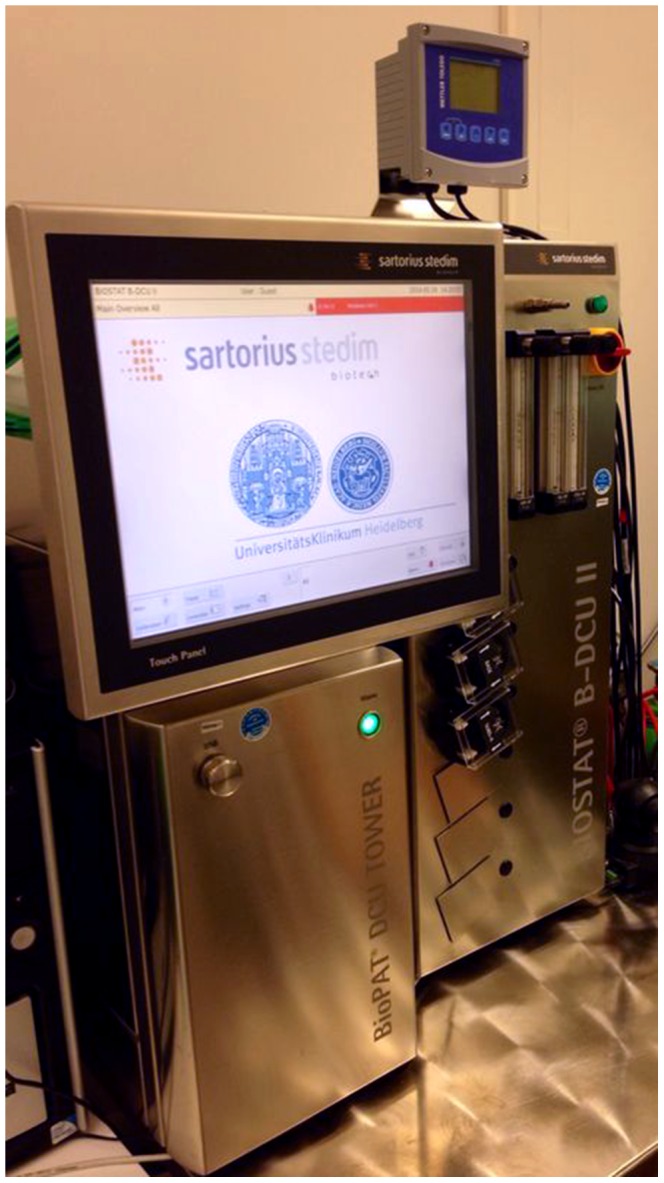
Whole-heart bioreactor: BIOSTAT B-DCU II and BioPAT DCU control tower (Sartorius Stedim Biotech GmbH, Germany).

Our bioreactor system is based on the BIOSTAT B-DCU II (Sartorius Stedim Biotech GmbH, Germany) which comprises a BioPAT DCU control tower (**Figure 1**) and a connected, custom-made culture-vessel for whole heart engineering. The culture-vessel is made of transparent glass, permitting direct observation, operated in a controlled milieu (37°C, 95% air, 5% CO2) with precise temperature- and pH-control. The system has a circulating volume of 5 L, with silicon-tubing connections, gas-ports for aeration, and provision for up to six peristaltic pumps for addition of corrective agents and/or volume-modulation. A high performance Servo-drive motor assembly combines low shear, gentle agitation for cell cultures and high speed mixing for microbial high cell density cultivation, ensuring high oxygen-transfer rates. The control system (DCU-4) ensures continuous, controlled perfusion flows and pressure.

### Murine neonatal cardiomyocytes

We harvested cardiomyocytes from freshly dissected ventricles of 1 to 3 day-old Sprague-Dawley rats (n = 30) using an isolation kit (Cellutron, Highland Park, NJ), promptly after euthanasia by decapitation. Cells were plated and cultured as described previously [14] in high-serum plating media (DMEM, 17% M199, 10% horse serum (HS), 5% fetal bovine serum (FBS), 100 U/mL penicillin and 50 mg/ml streptomycin) at 10,000 cells/cm^2^. After 18 hours, we transferred the cells to low serum maintenance media (DMEM, 18.5% M199, 5% HS, 1% FBS and antibiotics). Cell cultures were maintained at 37°C and 5% CO_2_ with addition of fresh maintenance media until optimized for injection, as confirmed by microscopy.

### Human umbilical cord-derived endothelial cells (HUVEC)

After review and approval by the ethical committee at the University of Heidelberg, we obtained written informed consent from 18 healthy pregnant women, procured umbilical cords immediately after delivery and stored them in PBS at 4°C. We cannulated the umbilical vein on both sides, flushed it with PBS, and filled it with 20 mL of 0.1% collagenase–dispase (Boehringer Mannheim, Mannheim, Germany) in Hanks' balanced salt solution (Gibco, Grand Island, NY, USA). After 20 min of incubation at 37°C, we gathered the cell suspension by rinsing the vein with 20 mL of medium 131 (Gibco, Grand Island, NY, USA) enriched with 10% FBS. Following centrifugation 1200 rpm for 10 minutes, we cultivated the clustered cells in 25-mL laboratory flasks under humidified incubator conditions until individual colonies coalesced. Finally, we confirmed the endothelial phenotype of the cultivated cells by microscopy and immunohistochemistry for expression of von Willebrand factor (vWF).

### Recellularization of decellularized porcine hearts

We mounted the decellularized porcine hearts in the bioreactor and perfused it at 37°C for 24 h at 100 mL/min with oxygenated cell-medium containing 1% gentamicin, to provide nutrients, ensure a physiological pH and achieve optimal conditions for recellularization. We then infused 5–6×10^6^ cells (HUVEC passage 3–6) via the aorta into the coronary arteries, temporarily interrupting the perfusion for 60 min to facilitate attachment of the HUVEC. For cell seeding, we delivered 8–9×10^6^ cells (neonatal cardiomyocytes) suspended in low serum maintenance media through five intramural injections (8–10 mm depth) of 200 µL each in the anterior left ventricle between the 1^st^ and 2^nd^ diagonal branch with a 27-G needle and a 1-mL tuberculin syringe.

### Organ perfusion of recellularized porcine hearts

We sutured sterile electrodes to the left midventricular wall, and paced the recellularized hearts using a Medtronic external pacemaker 5388 (Medtronic, Minnesota, USA). The culture medium in the bioreactor was replaced daily to replenish nutrients and eliminate waste products, with blood-gas analysis (Rapidlab 860, Siemens, Mannheim, Germany) every 12 h. We perfused with low serum maintenance media for 12–14 days. Four preparations were discontinued early because of infection (after 5, 7 and 11 days). The maximum perfusion time was 3 weeks, with maximum flows of 3.5 L/min. Following biomechanical assessment, we dissected the whole heart neoscaffolds, and sectioned and stained them as described before. As ‘control’ for comparison, we used decellularized but non-recellularized native-heart scaffolds, i.e. native-heart matrix treated identically, with the exception of the addition of cells.

### Live/Death Assay

We performed a live/death assay to assess the viability of reseeded cardiomyocytes in decellularized hearts after 7 days and 14 days in bioreactor culture. We washed tissue-bits in PBS thrice (3×5 min) and stained the tissue-bits with 2 mM calcein AM and 4 mM ethidium bromide (EthD-1) solution (live/dead assay; Invitrogen Corp. L3224) for 30 min at room temperature, according to the manufacturer's instructions. We then re-washed the tissue in PBS (3×5 min) and analyzed it by confocal microscopy.

### Evaluation of electrical activity of recellularized whole-heart neoscaffolds

For multi-electrode array (MEA) recordings after bioreactor conditioning, we took tissue bits from the injection area of the whole-heart neoscaffolds and fixed them in the center of the recording plates over a micro-drop of 0.1% gelatin. Electrical field potentials were recorded on a 60-channel amplifier (Multichannel Systems, Reutlingen, Germany), fed to threshold discriminators providing single event data for each spike, and analyzed. The sampling rate was 1.0 kHz, the cut-off frequencies of the filters were 0.5 Hz (high pass) and 1.0 kHz (low pass). We estimated the origin and direction of field potential propagation on the basis of latencies between electrical spikes at neighboring electrodes. For this purpose, we created event channels, taking the minimum of a spike as the event time. We then analyzed latencies between neighboring electrodes for ≥2 min of continuous recording to unequivocally identify the pacemaker site and the subsequent pathway of the spreading electrical signal.

### Statistical analysis

All values in the figures and text are shown as mean ± SEM. We performed a two-tailed independent Student's t-test to evaluate differences between native hearts (control) versus the decellularized hearts, considering p-values <0.05 statistically significant. SPSS 13.0 statistical software (SPSS Inc, Chicago, IL, USA) was used for data analysis.

## Results

### Perfusion-decellularization of porcine whole-hearts

**Figure 2:**
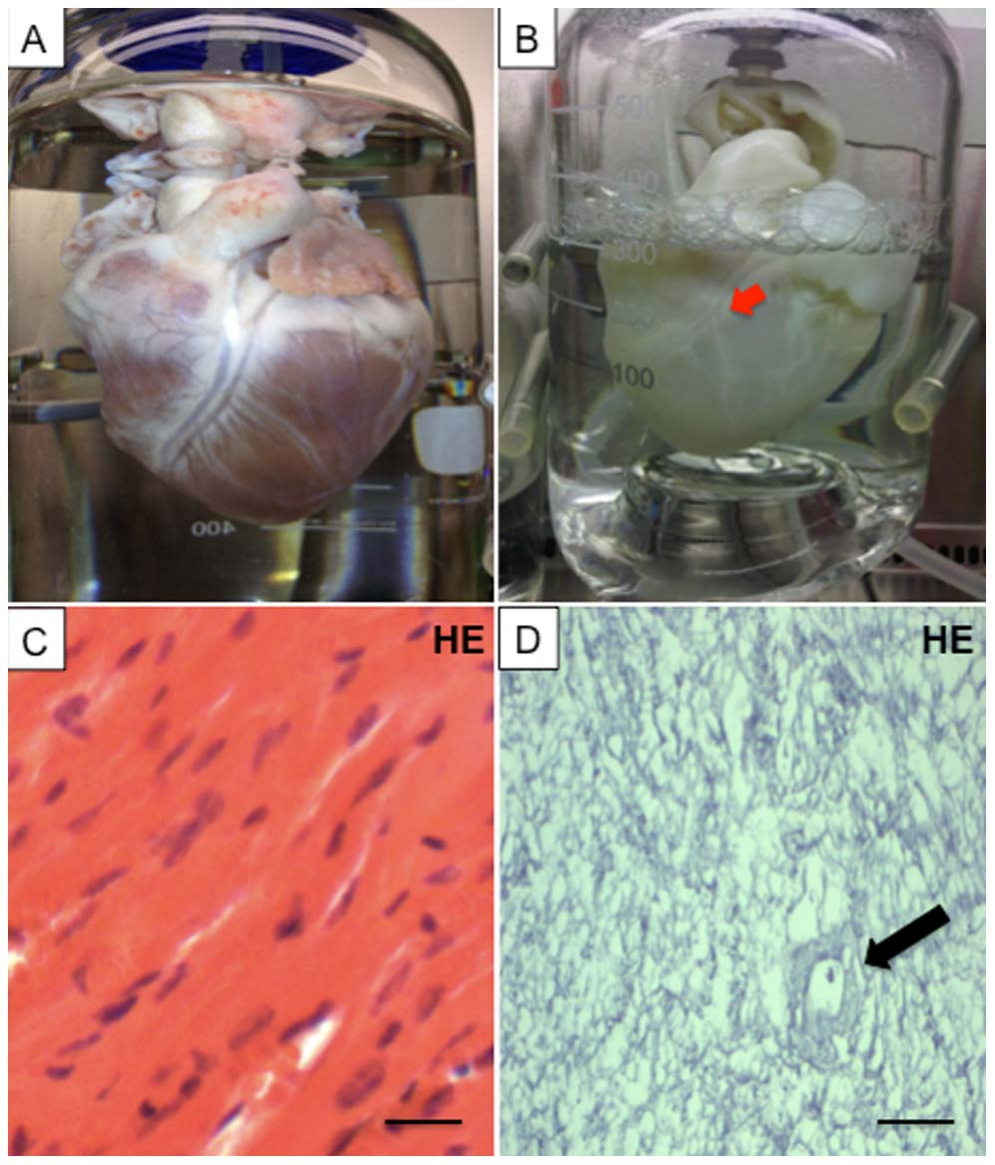
Representative images of a porcine heart before (A) and after (B) decellularization with sodium dodecyl sulfate (SDS). All structures including the coronary vasculature (B, red arrow) are preserved. Hematoxylin and eosin (HE) staining of ventricular tissue before (C) and after perfusion decellularization (D) showing no remnant nuclear structures after treatment with SDS, with maintained extracellular matrix and coronary vessels (D, black arrow). Scale bars, 200 µm.

Antegrade perfusion in the modified Langendorff model yielded fully decellularized porcine whole-hearts (**Figure 2A, 2B**). Histological evaluation revealed no remaining nuclei or contractile elements **(** **Figure 2C, 2D** **)**. The DNA content was significantly decreased after decellularization (473.2±13.4 ng DNA/mg tissue in the control group vs. 82.6±3.2 ng DNA/mg tissue in the decellularized group (p<0.05), whereas the glycosaminoglycan and elastin content were not significantly changed (**Table 1**).

**Table 1:** Results of Extracellular Matrix Analysis.

	Glycosaminoglycan Content	Elastin Content
Native Heart	4.61±0.15 µg	42.17±12.46 µg
Decellularized Heart	5.14±0.35 µg	23.66±3.28 µg

All values are expressed as mean ± SEM and adjusted to 1 mg lyophilized tissue sample.

### Properties of decellularized porcine whole-heart neoscaffolds

**Figure 3:**
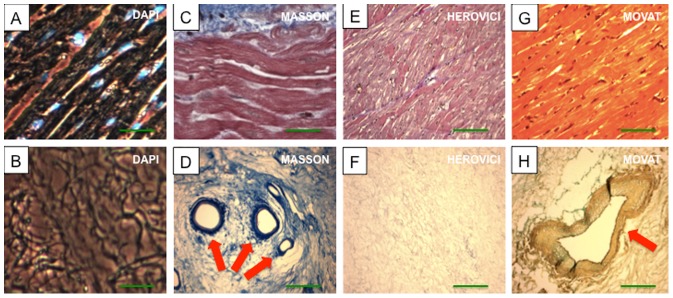
Images of native (A, C, E, G) and decellularized ventricular tissue (B, D, F, H) stained with DAPI, Masson's Trichrome Stain (Masson), Herovici's Stain (Herovici) and Movat's Pentachrome Stain (Movat) revealed absence of nuclear staining after decellularization (B) and stable preservation of extracellular matrix collagen, elastic fibers including large coronary vessels (D, H, red arrow) after the decellularization procedure. Scale bars, 200 µm.

**Figure 4:**
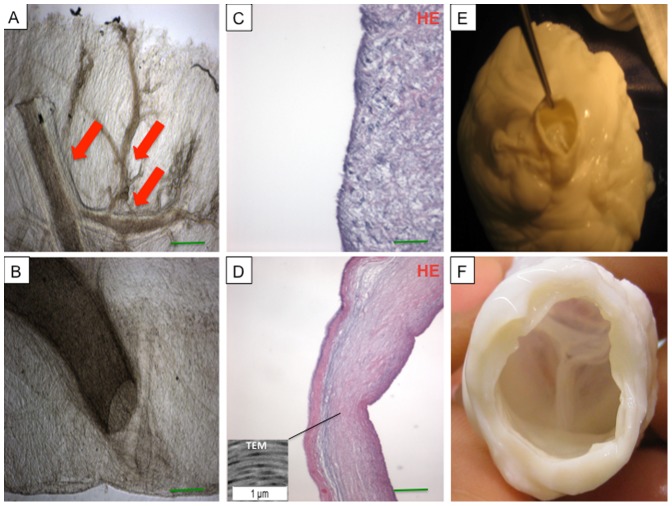
Photomicrographs of unstained tissue samples demonstrating intact coronary vasculature (A, B) with intact third- and fourth-level vessels (A, red arrows). The extracellular matrix composition of the aortic wall (**C**) and aortic valve leaflet (**D**) was preserved after decellularization and showed no remnant nuclear material as demonstrated by hematoxylin and eosin (HE) staining (C, D) and TEM analysis (**box in D**). Also the aortic valve remained competent after decellularization (**E, F**).

Following decellularization, the cardiac cells were removed from the hearts, but collagen types I and III remained, with preserved fiber composition and orientation of the myocardial ECM (**Figure 3** **A-H**). The vascular basal laminae were preserved within the retained ventricular ECM, bereft of endothelial cells or myocytes. Larger coronary vessels and the smaller third- and fourth-level branches remained patent (**Figure 4A, 4B**). Fiber orientation and composition was also preserved in the decellularized aortic wall and aortic valve leaflet as demonstrated by TEM analysis **(** **Figure 4C, 4D** **)**. The aortic valve remained competent (**Figure 4E, 4F**), as demonstrated by Evans blue perfusion studies.

**Figure 5:**
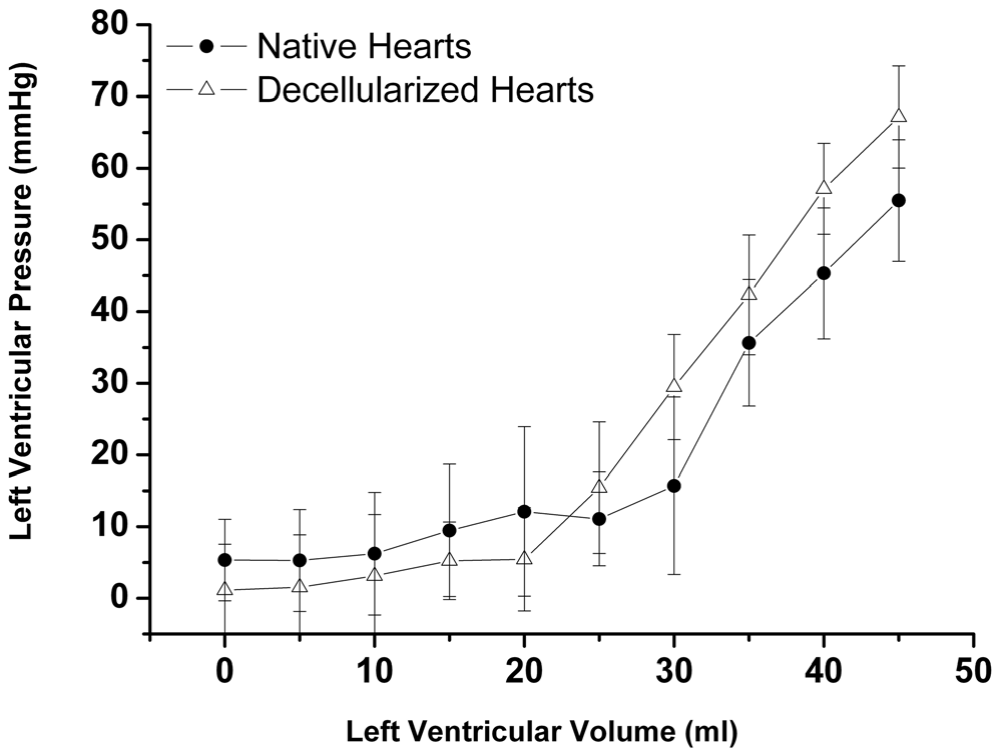
Results of biomechanical measurements. Left ventricular peak pressure vs. volume. Decellularized hearts showed similar mechanical stability as native hearts with no significant differences in biomechanical behavior. All values are expressed as mean ± SEM.

Results of the biomechanical tests are shown in **Figure 5**. Native and decellularized porcine hearts showed no major differences in biomechanical behavior, indicating mechanical/structural stability of the neoscaffold.

### Properties of recellularized porcine whole-heart neoscaffolds

**Figure 6:**
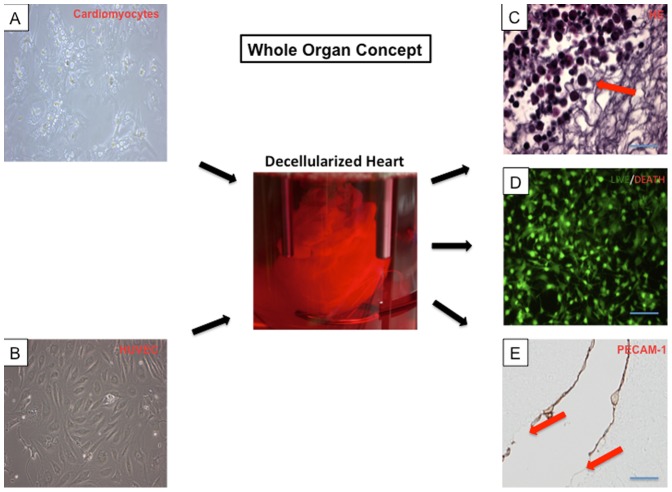
Whole organ concept. Cardiomyocytes **(A, 60× magnification)** and human umbilical vein endothelial cells (HUVEC) **(B, 60× magnification)** were reseeded in the decellularized porcine heart (center). Histological analysis demonstrated neonatal cardiomyocytes around the injection sites in the left ventricular wall **(C, red arrow)**. Live/death assay demonstrated viability of the reseeded neonatal cardiomyocytes in the cultured bioartificial hearts (**D**). A de novo layer of endothelial cells was generated, indicated by positive PECAM-1 staining (**E, cross section left coronary artery**). However, the new surface cell layer of endothelial cells was partially interrupted **(E, red arrows)**. Scale bars, 200 µm.

Histological analysis of recellularized porcine whole-heart neoscaffolds revealed a new layer of cobblestone-like cells in the coronary arteries, in both large and small coronary vessels, with partial interruption. Immunohistochemical interrogation identified the new surface cell layer as endothelial cells, confirmed by positive staining for PECAM-1 (**Figure 6E**). The native endothelial cell layer was reseeded by HUVEC (**Figure 6B**). The average recellularization with neonatal cardiomyocytes (**Figure 6A**) per cross-section of scaffold was more than 50% around the injection sites, with significant distal attrition. Recellularization was greatest in the area of injection (left ventricular mid-wall) (**Figure 6C**), compared to untreated areas (left ventricular apex and right ventricle). Live/death assay demonstrated preserved viability of the reseeded neonatal cardiac cells (**Figure 6D**).

**Figure 7:**
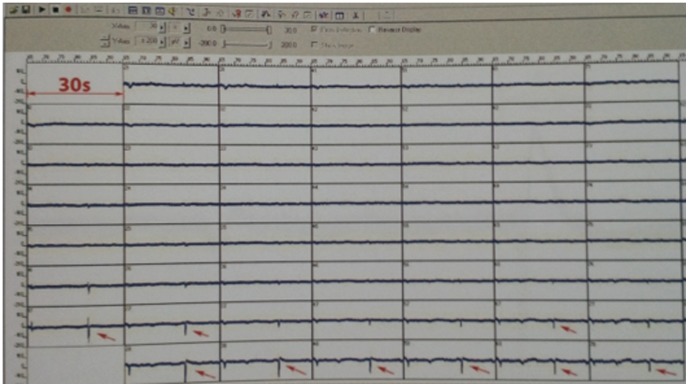
Demonstration of multi-electrode array electric voltage undulations of up to 200 mV in a time scale from ca. 500–1000 ms (red arrows) as a measure of myocardial electrical activity.

After ten days of bioreactor perfusion following cell seeding, the bioartificial hearts showed measurable electrical activity. MEA demonstrated discrete foci with electric voltage undulations of up to 200 mV in a time scale of ca. 500–1000 ms (**Figure 7**).

## Discussion

We present the first experimental prototype of a tissue-engineered human-sized porcine whole-heart, with perfused organ culture in a bioreactor and formation of myocardium that generates intrinsic electrical activity.

The term “Tissue Engineering” was introduced in 1987 by members of the US National Science Foundation (NSF) in Washington, D.C. and aims at generating functional 3D tissues outside of the body that can be tailored in size, shape, and function according to the respective needs before implanting them into the body [15]. Cardiac tissue engineering has historically involved several approaches: synthetic- or animal-derived extracellular matrix patches transplanted into small animals [16], cardiac myocyte culture with/without addition of extracellular matrix [17], and extraneous synthetic material as a scaffold for cell-seeding [18]. However, for cardiac tissue engineering to realize its full clinical potential, engineered tissues and organs must be structurally and functionally similar to healthy myocardium. Shortcomings with the previous approaches included unfavourable matrix properties such as limited diffusion capacity, low mechanical compliance, liberation of potentially toxic substances during degradation, and incompatibility with physiological cell growth [15]. Stacked cardiomyocyte sheets [19] have been used, but again, the creation of sufficiently thick cardiac sheets is limited by the inability to create the geometry necessary to support the high oxygen and energy demands of cardiomyocytes at thickness greater than ∼100 µm [20]– the “diffusion barrier”. Hence, tissue engineered myocardium requires elaborate ex vivo culture conditions.

Ott et al published their seminal work on perfusion-decellularization with cell-seeding in rat hearts [8], with subsequent progress by others [7], [9]. Whereas these experiments have undeniably been harbingers of progress in cardiac tissue engineering, murine hearts are much smaller in size and complexity than human hearts. On the other hand, porcine hearts are of a size comparable to human hearts, with similar physiology and anatomical features. A relatively short generation-interval of pigs means that a considerable number of bred porcine offspring can be procured in a reasonable time-period. Also, pigs have long been used in xenotransplantation studies, with genetically modified pigs shown to be significantly less susceptible to human complement-dependent cytotoxicity [21]. In addition, cardiac ECM exhibits remarkable homology between porcine and human hearts [22]. All these factors highlight the importance of porcine hearts in the attempt to develop a human-sized bioartificial heart.

Wainwright et al. [10] reported their work with porcine hearts, with decellularization accomplished by perfusion of hearts with different solutions and freezing at −80°C for cell lysis. We recently described a method for porcine heart decellularization that has the advantage of preserving biomechanical strength by minimizing damage to the ECM, with comparable reduction of cellular content using single-perfusion with SDS under constant pressure [11]. Whereas Wainwright et al [10] used sterilization pouches to reseed the surface of ECM sheets with subsequent lack of intrinsic electrical activity, our method preserves the 3D architecture of the entire heart, with transmural cardiomyocyte seeding and organ culture in a whole-heart bioreactor, with demonstrable myocardial electrical activity.

We used perfusion decellularization of porcine whole-hearts to develop a cardiac ECM scaffold with perfusable coronary vasculature, patent cardiac valves and intact three-dimensional architecture. The decellularized hearts showed similar mechanical stability as native hearts with no significant differences in biomechanical behavior. We could successfully repopulate the decellularized porcine hearts with murine cardiomyocytes and HUVEC. Whereas recellularization with cardiomyocytes was more evident in the areas of intramural injection, re-endothelialization was restricted to the coronary vasculature with delivery by perfusion, resulting in the loss of a significant number of cells in the effluent. Nevertheless, the native endothelial cell layer was reseeded by HUVECs, albeit with partial interruption. Although reseeding has been previously reported in rats [7]–[9], this is the first time that this technology has been scaled to whole hearts of human size and complexity. Porcine hearts, however, pose unique challenges. The costs involved are exponentially greater, translating into a relatively modest number of animals that can be procured concurrently. Whereas it would be ideal to reseed decellularized hearts with conspecific (from the same species) cardiac cells, the logistics of procuring porcine neonatal cardiomyocytes in amounts sufficient for reseeding are formidable and, presently, beyond our institutional resources. Nevertheless, it is encouraging that, despite reseeding with cardiomyocytes from a different/lower species, the decellularized porcine hearts exhibited measurable intrinsic electrical activity. Possibly, following reseeding with conspecific cardiomyocytes, a human-sized whole-heart neoscaffold may develop contractility and generate stroke work, evolving into a fully functional bioartificial heart as an alternative to transplantation. Presently, however, bioartificial heart transplantation is limited to heterotopic murine models, with perfused organ culture in the recellularized porcine model maintained for three weeks. Ongoing studies are directed at improving recellularization methods to enhance the dispersion/retention of reseeded cells, optimizing in-vitro strategies for organ maturation, and identifying stem/progenitor cells that can be harnessed for mass-production of autologous or off-the-shelf bioartificial solid organs for transplantation.

In conclusion, we present the first experimental prototype of a tissue-engineered human-sized porcine whole-heart, with perfused organ culture in a bioreactor and formation of myocardium that generates intrinsic electrical activity. Porcine hearts provide a promising tissue-engineering platform for the development of bioartificial hearts that may lead to future clinical strategies in the treatment of heart failure.

### Study Limitations

We could not obtain porcine neonatal cardiomyocytes for reseeding due to logistic/financial/ethical constraints and local government regulations. Xenocompatibility (species barrier) may affect the uptake and viability of murine cardiac cells and HUVECs reseeded in porcine hearts, which may have influenced the findings of this study.
